# The Uremic Toxin Indoxyl Sulfate Accelerates Thrombotic Response after Vascular Injury in Animal Models

**DOI:** 10.3390/toxins9070229

**Published:** 2017-07-19

**Authors:** Malgorzata Karbowska, Tomasz W. Kaminski, Natalia Marcinczyk, Tomasz Misztal, Tomasz Rusak, Lukasz Smyk, Dariusz Pawlak

**Affiliations:** 1 Department of Pharmacodynamics, Medical University of Bialystok, Mickiewicza 2C Str., 15-222 Bialystok, Poland; tomasz.kaminski@umb.edu.pl (T.W.K.); dariuszpawlak@poczta.onet.pl (D.P.); 2 Department of Biopharmacy, Medical University of Bialystok, Mickiewicza 2C Str., 15-222 Bialystok, Poland; nataliamarcinczyk@gmail.com; 3 Department of Physical Chemistry, Medical University of Bialystok, Mickiewicza 2A Str., 15-222 Bialystok, Poland; tomasz.misztal@umb.edu.pl (T.M.); tomasz.rusak@umb.edu.pl (T.R.); 4 Department of Pharmacology and Toxicology, University of Warmia and Mazury, Al. Warszawska 30, 10-082 Olsztyn, Poland; lukasz.smyk@gmail.com

**Keywords:** indoxyl sulfate, uremic toxin, thrombosis, hemostatic disorder, chronic kidney disease, prothrombotic state, cardiovascular event, tryptophan derivative

## Abstract

Chronic kidney disease (CKD) patients are at high risk for thrombotic events. Indoxyl sulfate (IS) is one of the most potent uremic toxins that accumulates during CKD. Even though IS is associated with an increased risk for cardiovascular disease, its impact on thrombotic events still remains not fully understood. The purpose of the study was to evaluate the direct effect of IS on thrombotic process. We examined the impact of acute exposure to IS on thrombus development induced by electric current in Wistar rats, intravital thrombus formation after laser-induced injury in the mice endothelium, coagulation profile, clot formation dynamics, platelet aggregations, and erythrocyte osmotic resistance. IS doses: 10, 30 and 100 mg/kg body weight (b.w.) increased weight of thrombus induced by electric current in dose-dependent manner (*p* < 0.001). Furthermore, two highest IS doses increased laser-induced thrombus formation observed *via* confocal system (increase in fluorescence intensity and total thrombus area (*p* < 0.01)). Only the highest IS dose decreased clotting time (*p* < 0.01) and increased maximum clot firmness (*p* < 0.05). IS did not affect blood morphology parameters and erythrocyte osmotic resistance, but augmented collagen-induced aggregation. Obtained data indicate that IS creates prothrombotic state and contributes to more stable thrombus formation. Thus, we concluded that IS may be one of crucial uremic factors promoting thrombotic events in CKD patients.

## 1. Introduction

Chronic kidney disease (CKD) is a pandemic problem with a global prevalence of 11–13% [1]. Patients suffering from CKD display a broad spectrum of hemostatic disorders leading on the one hand to increased bleeding tendencies, and to thrombosis on the other [2,3]. Generally, CKD is connected to an increased risk for thrombotic events, including incidence of cerebrovascular disease, myocardial infarction, and pulmonary embolism, as well as postangioplasty or stent thrombosis [4]. The etiology of spontaneous venous and arterial thrombosis in CKD patients is still not fully understood, however particular attention is currently being paid to the impact of accumulated toxins, which create the uremic *milieu*.

Indoxyl sulfate (IS) is one of the most potent uremic toxins that exerts aggressive and multi-directional impact on the body. It is tryptophan-derived compound, which levels can be even more than 80 times higher in patients with CKD compared to healthy people. Due to its high protein-bound ratio, presently there are no available methods for effective removal of IS [5]. IS is considered as a nephro-vascular toxin, which reduces production of nitric oxide and induces free radicals generation in a progressive manner [6,7]. Besides, it inhibits proliferation of endothelial and smooth muscle cells, and contributes to cardiomyocytes hypertrophy, myocardial fibrosis and aortic calcification [8,9]. Moreover, IS is a member of prothrombotic metabolites named “thrombolome”, which are considered as a novel risk factors for thrombosis [10]. Through its agonistic properties at aryl hydrocarbon receptor (AhR), IS regulates stability of tissue factor (TF) by inhibiting its ubiquitination and thereby enhances prothrombotic TF activity [11,12]. In addition, possibility of IS to promote thrombosis can be connected with the ability to induce phosphatidylserine exposure and microparticles release of red blood cells (RBCs) [13,14]. According to data provided by Ahmed et al., IS can trigger RBCs shrinkage and RBCs cell membrane scrambling that lead to suicidal erythrocyte death, which can impede microcirculation [13]. Furthermore, IS impairs endothelial progenitor cells, which lack is connected with thrombotic events after endovascular interventions [15,16]. In recent times, higher level of IS was connected with increased risk for dialysis graft thrombosis and associated with higher number of thrombectomy procedures [17,18]. 

However, despite that some studies show correlation between IS and thrombosis in hemodialysis patients [17,18], the probable mechanism connecting IS with thrombotic events during CKD still remains unclear. Currently, there is only one available in vivo study demonstrating IS prothrombotic property in animal model, precisely FeCl3-induced carotid artery thrombosis model in mice [19]. Taking above into consideration, the aim of the present study was to evaluate direct effect of IS on the hemostatic system and thrombotic process in various independent models: in vitro, in vivo and ex vivo. We examined the impact of acute exposure to IS on (1) thrombus development induced by direct electric current in Wistar rat’s model; (2) intravital thrombus formation after argon-ion laser-induced endothelial injury in the mesenteric vessels of mice; (3) coagulation profile; (4) dynamics of clot formation; (5) platelet aggregations in different in vitro models; as well as (6) osmotic resistance of RBCs.

## 2. Results

### 2.1. The Concentrations of IS in the Rats Plasma

**Figure 1:**
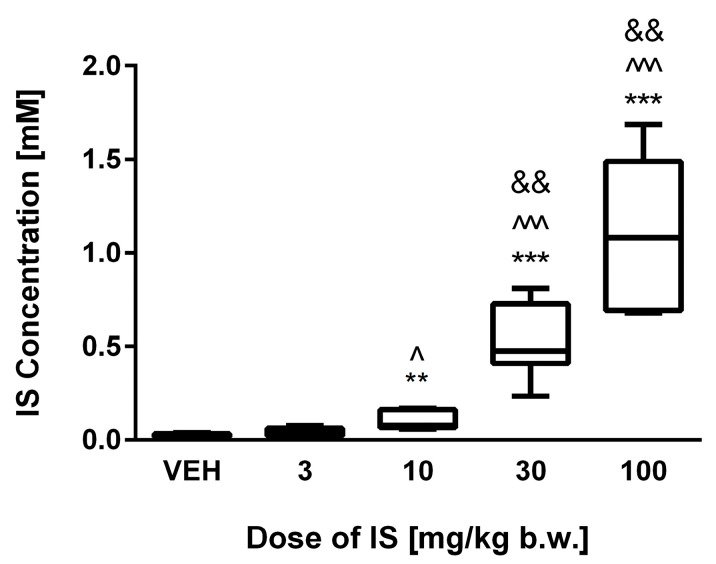
The plasma concentrations of IS following acute administration. VEH—control group; IS—indoxyl sulfate; ** *p* < 0.01 compared to control; *** *p* < 0.001 compared to control; ^^^ *p* < 0.05 compared to 3 mg/kg body weight (b.w.) of IS; ^^^^^ *p* < 0.001 compared to 3 mg/kg b.w. of IS; ^&&^ *p* < 0.01 compared to 10 mg/kg b.w. of IS.

As shown in Figure 1, acute administration of IS in the doses of 10 mg/kg body weight (b.w.) (*p* < 0.01), 30 mg/kg b.w. (*p* < 0.0001), and 100 mg/kg b.w. (*p* < 0.0001) led to statistically significant increase in IS concentrations compared to controls. Only the lowest dose of 3 mg/kg b.w of IS did not statistically influenced the concentration of IS, however trend for increased values was observed (*p* = 0.053). Moreover, the highest IS dose significantly increased IS concentration compared to 10 mg/kg b.w. (*p* < 0.01) and 3 mg/kg b.w. (*p* < 0.001). In turn, the IS dose of 30 mg/kg b.w. significantly augmented IS concentration compared to IS doses 10 and 3 mg/kg b.w. (both *p* < 0.01). IS in the dose of 10 mg/kg b.w. also increased IS concentration compared to 3 mg/kg b.w. (*p* < 0.05).

### 2.2. Impact of IS on Thrombus Development in Rats Model of Arterial Thrombosis

**Figure 2:**
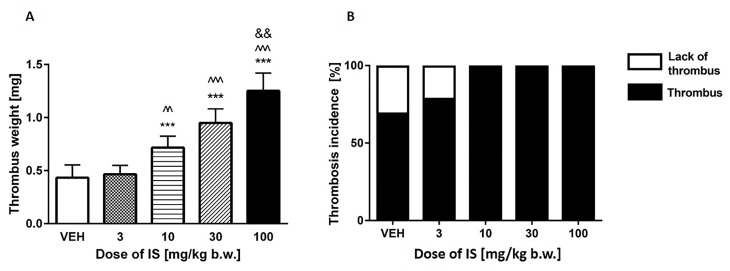
(**A**) The weight of the developed thrombus after exposure on IS in arterial thrombosis model; (**B**) Frequency of the thrombotic incidence. VEH—control group; IS—indoxyl sulfate; *** *p* < 0.001 compared to control; ^^^^ *p* < 0.01 compared to 3 mg/kg b.w. of IS; ^^^^^ *p* < 0.001 compared to 3 mg/kg b.w. of IS; ^&&^ *p* < 0.01 compared to 10 mg/kg b.w. of IS.

IS doses of 10, 30, and 100 mg/kg b.w. showed statistically significant increase in the arterial thrombus weight in a dose-dependent manner (*p* values respectively: <0.01; <0.001; <0.001 compared to control group) (Figure 2A). The lowest dose of IS did not change the weight of the thrombus. Furthermore, the highest IS dose significantly increased thrombus weight compared to IS doses 3 mg/kg b.w. (*p* < 0.001) and 10 mg/kg b.w. (*p* < 0.01). In turn, IS doses 10 and 30 mg/kg b.w. augmented the weight of thrombus compared to the dose of 3 mg/kg b.w. of IS (*p* < 0.01; *p* < 0.001, respectively). The parameter of thrombosis incidence (Figure 2B) showed that doses, which statistically increased the thrombus weight also led to increase in thrombosis incidence frequency with 100% ratio. In control group the thrombosis incidence has occurred in 61.5% of procedures, and in group that were treated of 3 mg/kg b.w. of IS with the 75% frequency. 

### 2.3. Effects of IS on Thrombus Formation after Laser-Induced Vascular Injury in Mice: Intravital Imaging

**Figure 3:**
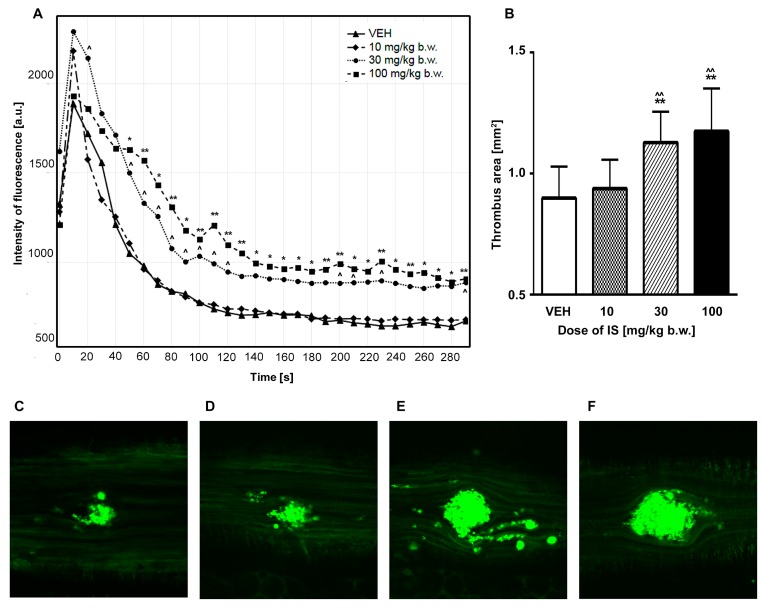
(**A**) Impact of IS on fluorescence intensity in intravital imaging of the thrombus development during 5 min evaluation after laser injury in mice model; (**B**) Effect of IS on thrombus area after laser-induced thrombus formation in intravital mice model; (**C**–**F**) Representative images of the developed thrombi in intravital model after administration of 0.9% natrium chloride (control group) or IS; water immersion, ×200. (**C**) VEH; (**D**) 10 mg/kg b.w. of IS; (**E**) 30 mg/kg b.w. of IS; (**F**) 100 mg/kg b.w. of IS. IS—indoxyl sulfate; VEH—control group; * *p* < 0.05 compared to control; ** *p* < 0.01 compared to control; ^^^ *p* < 0.05 compared to the dose of 10 mg/kg b.w. of IS; ^^^^ *p* < 0.01 compared to the dose of 10 mg/kg b.w. of IS.

As shown in Figure 3B, the two highest doses of IS (30 and 100 mg/kg b.w.) led to accelerated thrombus formation in the area of the laser-injured endothelium in mice. IS in doses of 30 and 100 mg/kg b.w. caused statistically significant (both *p* values < 0.05) augmentation of thrombus size compared to control group. The same doses significantly increased thrombus area compared to the doses of 10 mg/kg b.w. of IS (*p* < 0.01). Moreover, the progression of thrombus development was more dynamic after administration of these IS doses that is reflected in statistically significance changes of fluorescence intensity in the vast majority of the studied time points compared to control group (Figure 3A). The dose of 10 mg/kg b.w. did not affect these parameters. Figure 3C–F show representative images of thrombi formed after administration of different IS doses or 0.9% natrium chloride (in control group).

### 2.4. Evaluation of IS Impact on Coagulation Parameters

The doses of 10, 30, 100 mg/kg b.w. led to significantly decreased values of activated partial thromboplastin time (APTT) (all *p* < 0.001) and concentration of fibrinogen (*p* < 0.05, *p* < 0.01, *p* < 0.05, respectively). Moreover, only the dose of 10 mg/kg b.w. led to increase in prothrombin time (PT) (*p* < 0.05). None of the used IS doses affected thrombin time (TT) (Table 1).

### 2.5. Thromboelastometric (ROTEM) Analysis of the Effect of IS on Dynamics of Clot Formation

**Figure 4:**
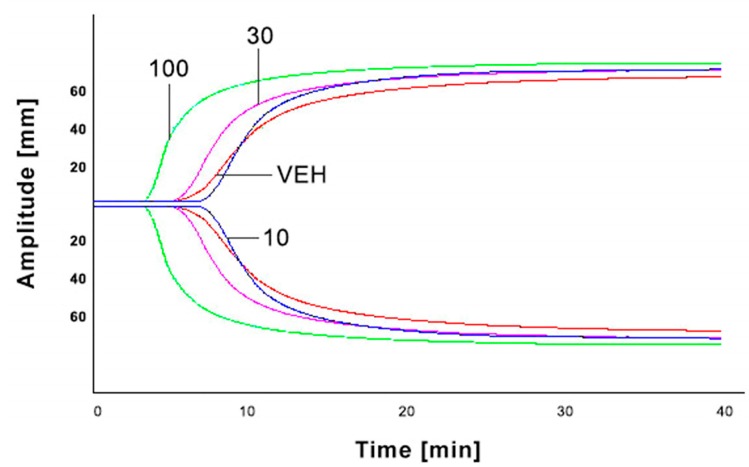
Graphical representation of IS impact on ROTEM analyses. IS—indoxyl sulfate; VEH—control group; 10—the dose of 10 mg/kg b.w. of IS; 30—the dose of 30 mg/kg b.w. of IS; 100—the dose of 100 mg/kg b.w. of IS.

Only the highest dose of IS (100 mg/kg b.w.) statistically shortened clotting time (CT) with *p* < 0.01 compared to control group, with *p* < 0.01 compared to the dose of 10 mg/kg b.w. and with *p* < 0.05 in comparison to 30 mg/kg b.w. Besides, the same highest IS dose shortened clot formation time (CFT) (*p* < 0.05 compared to controls). Moreover, the same dose of IS increased the total area under the curve (AUC) (*p* < 0.05 compared to control group) and maximal clot firmness (MCF) parameter (*p* < 0.05 in comparison to controls and IS doses of 10 and 30 mg/kg b.w.). We did not observe any significant changes in alpha angle values after administration of IS doses (Table 2, Figure 4).

### 2.6. Fibrin Generation Assay

**Figure 5:**
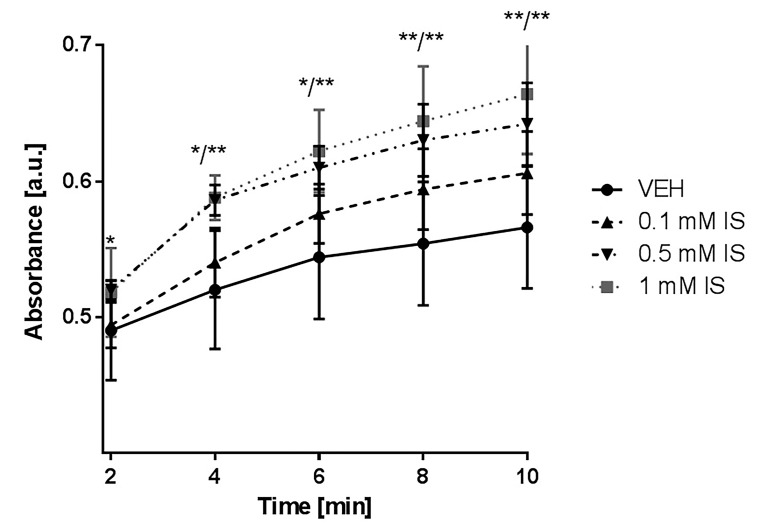
Impact of IS on fibrin generation measured in platelet-depleted plasma. IS—indoxyl sulfate; VEH—control group; * *p* < 0.05 compared to control; ** *p* < 0.01 compared to control.

The highest concentration of IS 1 mM significantly augmented fibrin generation compared to control that was reflected by the strongest absorbance. Moreover, the concentration of 0.5 mM also affected fibrin generation by increasing it (Figure 5).

### 2.7. Influence of IS on In Vitro Aggregation in Whole Blood and Platelets Rich Plasma (PRP)

**Figure 6:**
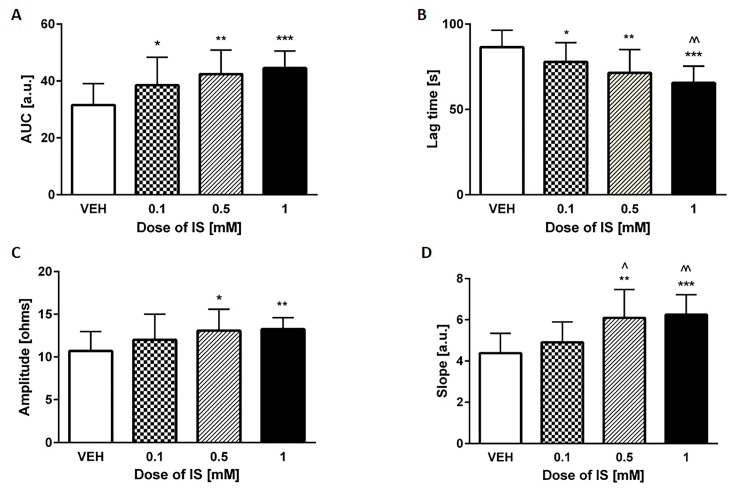
Effect of IS on collagen-induced in vitro aggregation in whole blood. (**A**) area under the curve (AUC); (**B**) lag time of aggregation; (**C**) amplitude of the aggregation; (**D**) slope. IS—indoxyl sulfate; VEH—control group; * *p* < 0.05 compared to control; ** *p* < 0.01 compared to control; *** *p* < 0.001 compared to control; ^^^ *p* < 0.05 compared to 0.1 mM of IS; ^^^^ *p* < 0.01 compared to 0.1 mM of IS.

As shown in Figure 6, both values the amplitude and the slope were affected by two highest concentrations of IS (0.5 and 1 mM). Concentration of 0.5 mM increased amplitude with *p* < 0.05 and the slope with *p* < 0.01, whereas 1 mM of IS augmented the amplitude with *p* < 0.01 and the slope with *p* < 0.001. Moreover, all of the tested doses (0.1, 0.5, and 1 mM) changed the AUC and lag time of aggregation (with *p* values of: <0.05, <0.01 and <0.001, respectively, for both parameters). Besides, concentration of 1 mM decreased lag time compared to concentration of 0.1 mM of IS, and two highest concentration of IS 0.5 and 1 mM increased slope compared to 0.1 mM of IS (*p* < 0.05, *p* < 0.01, respectively).

None of the measured parameters in optical PRP aggregation stimulated by adenosine diphosphate (ADP) and ADP with serotonin (5-HT) were affected by used IS concentrations (Table 3).

### 2.8. Impact of IS on Erythrocyte Osmotic Resistance

**Figure 7:**
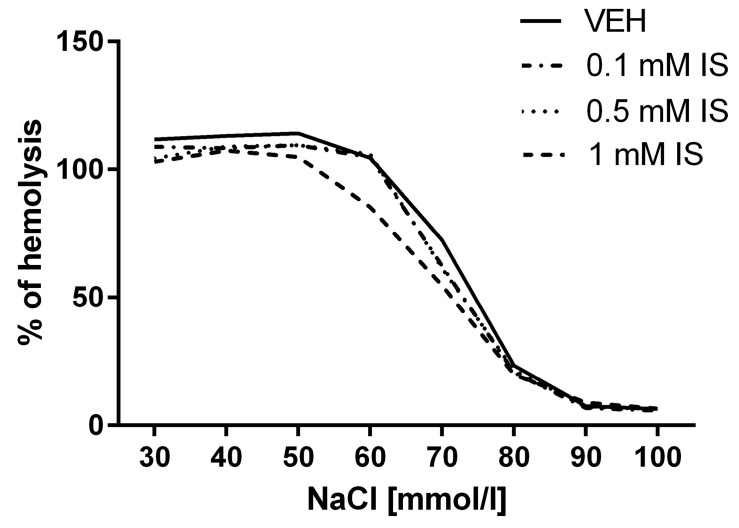
Impact of IS on osmotic resistance of erythrocytes reflected as % of hemolysis.

None of the used concentrations of IS (0.1, 0.5, and 1 mM) had an impact on the susceptibility to hypotonia-induced lysis of RBCs (Figure 7).

### 2.9. Effect of IS on Hematological Parameters

None of the tested IS doses (3, 10, 30 and 100 mg/kg b.w.) affected hematological parameters like hematocrit (HCT), hemoglobin (HGB), mean corpuscular hemoglobin (MCH), mean corpuscular hemoglobin concentration (MCHC), mean corpuscular volume (MCV), platelets (PLT), RBCs, and white blood cells (WBC) (Table 4).

## 3. Discussion

In the present study, we found that IS accelerates thrombotic response to vascular injury. To our knowledge, this is the first study demonstrating direct impact of IS on the thrombotic process in rats and mice models. We confirmed prothrombotic effect of the mentioned toxin in two independent animal models of thrombosis—(1) model of arterial thrombosis induced by direct electric current in Wistar rats and (2) after laser-induced injury in the endothelium of mice using confocal and widefield intravital microscopy. Moreover, we also confirmed in ex vivo and in vitro models that IS activity is directed toward creating prothrombotic state and mentioned toxin leads to more stable clot formation. Obtained data indicate that IS is certainly one of the crucial uremic factors promoting thrombotic events.

CKD is an independent and well-established risk factor for cardiovascular diseases (CVD), and what is more, the risk for cardiovascular events increases with the progression of CKD [6]. Progression of CKD due to reduced renal clearance also results in accumulation of various toxins like IS [5]. Increased concentration of IS has been found to be associated with numerous CVD in CKD patients such as arteriosclerosis, coronary heart disease and peripheral artery disease [4,20]. Regarding thrombotic events, IS is currently associated with increased risk for postangioplasty dialysis graft thrombosis [18]. In addition, Lin et al. [17] showed that IS level is positively correlated with a number of thrombectomy procedures in hemodialysis patients. Plasma concentration of IS in pre-dialysis CKD patients can be even higher than 1 mM (mainly in stages IV/V and end-stage renal disease) and due to high protein-bound ratio of IS (approximately 90%), its reduction rate after hemodialysis is only about 30% [5,6]. Thus, doses or concentrations used in our study reflect level of both pre-dialysis and hemodialysis patients (Figure 1). Moreover, the lowest dose of IS used by us reflects level of IS in CKD patients on conservative treatments that are not qualified for renal replacement therapy. Therefore, observed effects of IS can concern each groups of CKD patients.

The present study demonstrates in various models that IS is able to increase the thrombus weight and its total area, as well as augmented dynamics of formation and stability of clots (Figure 2, Figure 3 and Figure 4, Table 2). In case of laser-induced vascular injury model, coagulation is initiated by TF and platelets are activated mainly through TF-mediated thrombin generation. Moreover, in this model TF is mostly derived from circulating blood [21]. Many researchers documented that IS influences activity of TF. In endothelial and peripheral blood mononuclear cells IS contributes to increased TF production and Gondouin et al. [22] reported that mechanism of augmented TF transcription in this case was connected with activation of AhR-related toxicity pathway. In turn, in smooth muscle cells IS prolonged TF activity by suppressing its ubiquitination [11,12]. Moreover, Yisireyili et al. [23] suggested that IS induces activation of (pro)renin receptors in human aortic smooth muscle cell through AhR/nuclear factor-κB p65 pathway that also results in stimulation of TF expression. In addition, our previous research showed that level of IS positively correlated with TF in CKD patients. Therefore, in cases of vascular injury or plaque rapture, resulting in exposure of TF (especially monocytic TF), IS can accelerate formation of thrombus, particularly after stent implantation.

Alteration in hemostasis in CKD can result in excessive or attenuated coagulation, and the number of hemostatic disturbances increases with reduction of excretory kidney function. Available data indicate that CKD patients reveal higher levels of fibrinogen and coagulation factors, like factor VII, VIII, XIII, and von Willebrand factor (marker of endothelial damage), as well as indicator of in vivo thrombin generation—prothrombin fragments F_1+2_ [2]. In our previous study, we have noticed correlation between IS level and hemostasis-related parameters such as von Willebrand factor, soluble urokinase-type plasminogen activator receptor and soluble cell adhesion molecule (sICAM-1), which, in turn were positively associated with CVD prevalence in CKD patients. Moreover, we have linked IS with hemostatic disorders through its ability to intensify oxidative stress and inflammation, and to affect monocyte activation and transition [24]. Our present research demonstrates that IS led to decreased concentration of fibrinogen (Table 1). It can be explained by the depletion caused by augmented fibrin generation that we observed via turbidimetric method (Figure 5). Besides, we observed shortened APTT (Table 1) that is considered as a risk factor for venous thromboembolism. Furthermore, we did not observe significant changes in PT after higher doses of IS, which can suggest that IS can influence coagulation cascade mainly through factors connected with intrinsic pathway (Table 1). Shortened time of blood clotting by IS was confirmed not only by APTT, but also by decrease in CT and CFT (Figure 4, Table 2)—parameters obtained due to thromboelastometry [25]. Using ROTEM technique we obtained information about both initiation and overall dynamics of clot formation, which indicates that IS contributes to increased clot firmness as well as augmented speed of solid clots formation (Figure 4, Table 2). 

Furthermore, our results showed that IS increased thrombus weight in a dose-dependent manner in a model of electric current-induced thrombosis. What is important, we observed increase in thrombosis incidents frequency with 100% ratio accompanying increase in thrombus weight (Figure 2). Mentioned model applies to arterial thrombosis in which platelets play crucial role. According to data from Guarini [26], thrombus in this model is a mix of leukocytes and erythrocytes, and consists largely of platelet aggregation and fibrin with trapped erythrocytes and leukocytes. In turn, in a model of thrombosis induced by laser injury in the mesenteric vessels of a living mouse, IS significantly increased platelet accumulation in area of endothelium laser injury and augmented total thrombus area, which was reflected by intensified fluorescence (Figure 3). Overall, baseline platelets reactivity in patients with CKD is increased [3]. Taking this into account, we evaluated impact of IS on platelet aggregation. Addition of IS solely into PRP or whole blood did not affect platelet aggregation, which indicates that IS is not an aggregation inductor (data not shown). Moreover, obtained data showed that IS did not affect ADP, ADP+ 5-HT (Table 3) and collagen-induced aggregation in PRP and ADP-induced aggregation in whole blood (data not shown). Whereas addition of IS exacerbated collagen-induced platelet aggregation in whole blood (Figure 6). Observed results suggest that accelerated thrombotic response caused by IS is not a result of its direct impact on platelets, but rather on other components like RBCs. In another study, Yang et al. did not observe increase in the expression level of platelet activation marker—P-selectin in the resting platelets after administration of IS to mice, but observed its augmented level in platelets after IS administration and collagen stimulation [19]. Erythrocytes enhance collagen-induced platelet recruitment and responsiveness [27]. Furthermore, RBCs contribute to increased expression of P-selectin on platelets [28]. Mechanism of laser-induced thrombosis is also connected with P-selectin expression on the surface of platelets [20], therefore we suppose that noticed increased platelet accumulation in thrombus area is not a results of direct IS effect on platelets.

Due to the observed augmentation of collagen-induced platelet aggregation in whole blood, we decided to examine the IS impact on osmotic resistance of RBCs. IS did not influence osmotic fragility of RBCs (Figure 7) that is consistent with data presented by Ahmed et al. [13]. They demonstrated that IS triggers phosphatidylserine exposure at the RBCs without changing permeability of its membrane to hemoglobin. In addition, exposure to IS promoted erythrocyte shrinkage and cell membrane phospholipid scrambling that also led to suicidal death of erythrocytes–eryptosis [13,14]. Probable mechanism of this action includes IS-induced increase in cytosolic Ca^2+^ concentration and enhanced ceramide formation. Besides, eryptosis can be triggered by other factors like oxidative stress or p38 MAP kinase that can be induced by IS [14]. Acceleration of RBCs deaths contributes to impaired microcirculation not only via adherence of phosphatidylserine-exposing RBCs to the vascular wall, but also through releasing microparticles from erythrocytes [14,29]. Generally, RBCs microparticles promote generation of thrombin through activation of factor XII [30,31]. These data suggest that IS can induce thrombin generation in this manner, which is also in line with our observation of IS-induced activation of intrinsic pathway of coagulation, reflected by decreased APTT (Table 1). Besides, microparticles provide a membrane surface for assemble of coagulation factors and some types of microparticles contain TF that facilitates thrombus formation. IS also induces release of microparticles originated from cells different than RBCs like endothelial cells, which can trigger endothelial cell thrombogenicity via activation of the mitogen-activated protein kinases and PI3-kinase pathway mediated by angiotensin II/angiotensin II receptor type 1/nicotinamide adenine dinucleotide phosphate-oxidase signaling [31,32]. Beyond this, microparticles can be released by monocyte/macrophages during inflammatory state. It is not excluded that IS, which promotes inflammatory state and differentiation of monocyte into macrophages with pro-fibrotic phenotype, can also enhance release of this kind of microparticles [33,34].

## 4. Conclusions

In conclusion, this is the first study providing evidence that IS contributes to increased thrombus formation in various independent animal models. We demonstrated that acute exposure to IS accelerates thrombotic response after vascular injury and promotes more stable clot formation. The strongest of our study is the fact that used models allow solely to observe the effect of IS without interferences of other toxins creating uremic *milieu*. Despite we did not point precise mechanism of described effects and we did not clarify role of TF in our observation, we hypothesize that IS due to its procoagulant activity is one of the crucial uremic factors promoting thrombosis in patients suffering from CKD. The observed results confirm that IS is rightly included to “thrombolome” group, and developing methods for its effective removal may result in a better prevention of thrombotic events in CKD patients. However, the model used in presented work does not demonstrate all conditions of CKD, thus other studies with CKD models are necessary to fully understand thrombotic process in CKD patients. Furthermore, in the view of complexity of CKD and a number of uremic toxins, effects of other gut-derived uremic toxins including p-cresol should also be examined.

## 5. Materials and Methods

### 5.1. Animals

**Scheme 1:**
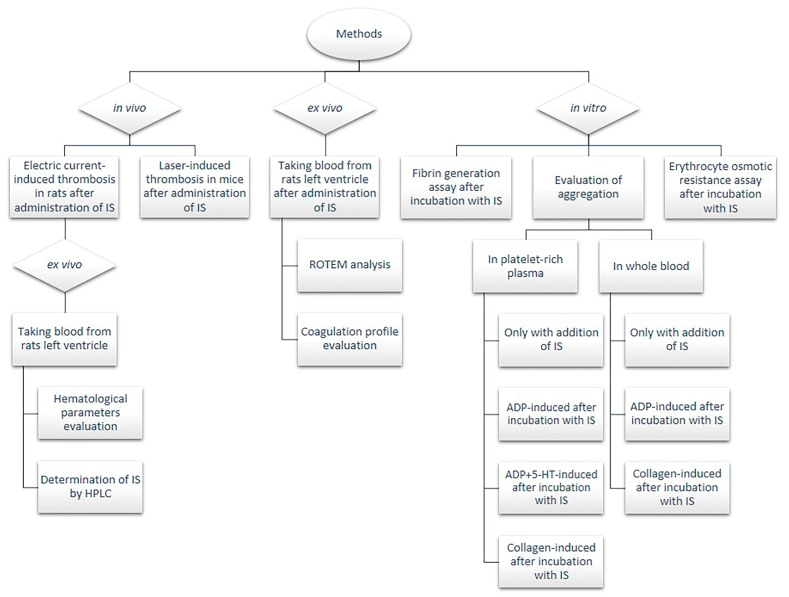
Flow-chart of experimental procedures. 5-HT—serotonin; ADP—adenosine diphosphate; HPLC—high performance liquid chromatography; IS—indoxyl sulfate; ROTEM—rotational thromboelastometry.

Eight-week old male Wistar Crl:WI (cmdb) rats weighting 220–240 g and four-week old male C57BL6/cmdb mice weighting 20–23 g purchased from the Centre for Experimental Medicine of Medical University of Bialystok were maintained under standard conditions at 22–25 °C with a 12 h day/night cycle and with free access to standard rats/mice chow and tap water *ad libitum*. Before initiating the experimental procedures, animals were fasted for 24 h, but were allowed free water access. All procedures (Scheme 1) involving animals were approved by a Local Bioethics Committee (Agreement No. 124/2015; Date of approval: 1 July 2015) and conducted in accordance with the institutional guidelines.

### 5.2. Determination of IS in Plasma

Concentration of IS in the plasma was evaluated using liquid chromatography with fluorescence detection according to Al’Zhabi et al. [35] with further modification. Determination of IS was carried out at temperature of 24 °C. The chromatographic equipment was an Agilent 1200 series LC-system (Agilent Technologies, Boblingen, Germany) composed of G1322A degasser, G1311A quaternary pump, G1329A autosampler and G1330B thermostat for autosampler, HP1046A fluorescence detector (FLD). Deproteinated samples were prepared by adding 0.4 mL acetonitrile containing the methyl paraben (1 mg/mL) as internal standard into the 0.1 mL plasma. The samples were vortexed, kept at 4 °C for 1 min, and then centrifuged for 30 min 14,000 *g* at 4 °C. Further, 1 mL of the supernatant was injected into high performance liquid chromatography (HPLC) system for analysis. The prepared samples were separated on column Phenomenex PEPTIDE 3.6 mm XB-C18 4.6 × 250 mm (Phenomenex, Torrance, CA, USA). The column effluent was monitored by using programmable FLD. The optimized conditions were determined by recording fluorescence spectra with a stop-flow technique. Excitation and emission wavelengths were set at 280/375 nm. The output of the detector was connected to a single instrument LC ChemStation. The mobile phase was composed of acetate buffer (pH 4.5) containing 90% of acetonitrile and it was pumped at a flow-rate of 0.8 mL/min.

### 5.3. Rat Model of Arterial Thrombosis Induced by Direct Electric Current

Arterial thrombosis induced by direct electric current was described previously by Schumacher et al. [36]. Rats were anesthetized with pentobarbital (40 mg/kg b.w., ip) and IS was administered iv in one of four doses: 3, 10, 30 or 100 mg/kg b.w. into left femoral vein 10 min before induction of thrombosis. In control group 0.9% natrium chloride was administered. Thrombus development was induced by 10 min applying a constant electrical current (10 mA) to the external surface of the right carotid artery using the anode and the cathode attached subcutaneously to the hind limb. Animals were kept on a heated table to avoid chilling and loss of temperature. After 60 min of the stimulation, the thrombus was taken out, air-dried (24 h) and weighed using high-sensitive weighing scales.

### 5.4. Intravital Imaging of Thrombus Formation after Laser-Induced Vascular Injury in Mice

Intravital imaging was based on methods described by Falati et al. [37] with further modifications. Fixed-stage microscope Zeiss Axio Examiner Z.1 (Carl Zeiss Microscopy, Jena, Germany) was used to intravital observation of thrombus development at the site of laser-induced endothelial injury in the mouse mesentery. Wild type mice were anesthetized with ketamine and xylazine (120 mg/kg b.w., ip, 12.5 mg/kg b.w., ip). IS (10, 30 or 100 mg/kg b.w.) in 0.1 mL of 0.9% natrium chloride was administrated into left femoral vein 60 min before laser injury. 0.1 mL of 0.9% natrium chloride served as a control. DiOC6(3) (0.1 mM in 0.05 mL of the mixture of DMSO and PBS; volume ratio 1:50) was injected into right femoral vein 10 min before laser injury. A midline laparotomy incision was made, and then the mesentery of the ileum was pulled out of the abdomen and draped over a plastic mound. The mesentery was continuously perfused with 37 °C-warmed saline to prevent the vessels from drying. The mesentery was placed under the objective (W Plan-Apochromat 20×/1.0 water immersion objective, Carl Zeiss Microscopy, Jena, Germany) and identified. During the experiment DiOC6(3) was excited by 488 nm laser (LaserStack 488 nm, 3iL33, Intelligent Imaging Innovations—3I, Denver, CO, USA) to visualize thrombus development. Endothelial injury was induced by pulsed 532 nm argon-ion laser beam (Ablatel, 3iL33, Intelligent Imaging Innovations, Inc., Denver, CO, USA) aimed through the microscope objective lens. The thrombus progression was registered for 5 min with Confocal Scanner Unit CSU-X1 (Yokogawa Electric Corporation, Tokyo, Japan) in one focal plane (2D imaging) corresponding with the largest area of the thrombus. At most six thrombi were induced in one mouse at particular time points. Collected images (four images per second) were analysed using Slide Book 6 (Intelligent Imaging Innovations, Denver, CO, USA). The regions of thrombus fluorescence were encircled every 5 s of record in particular image. The areas of regions from one thrombus were calculated with SlideBook6 and then added up.

### 5.5. Evaluation of Coagulation Profile

APTT, PT and TT were automatically determined by chronometric method (coagulometer Coag-Chrom 3003, BioKsel, Grudziadz, Poland) using standard laboratory reagents in previously collected rats’ plasma. The fibrinogen concentration was evaluated according to the Clauss method using the same coagulometer and commercial kit provided by BioKsel, Poland.

### 5.6. Fibrin Generation Assay

Fibrin generation curves were evaluated by turbidimetric method according to Bjornsson et al. [38] in plasma samples at 650 nm, 25 °C after 45 min incubation with 0.1 mM, 0.5 mM, and 1 mM concentrations of IS or 0.9% natrium chloride that served as a control.

### 5.7. ROTEM Analyses

The evaluation of the impact of IS on clot stability and elasticity was performed using ROTEM technology described previously by Luddington [39] on a blood withdrawn from the rats left ventricle on a 3.13% sodium citrate after IS administration (10, 30, or 100 mg/kg b.w. iv). 0.9% natrium chloride served as a control. Measurements were performed using ROTEM system (Tem International GmbH, Mannheim, Germany) as described in details in Misztal et al. [40]. To evaluate dynamics of clot formation, the following parameters were measured: CT—time from start of measurement to the beginning of the fibrin polymerization process; CFT—time from start of measurement to stable clot formation; alpha angle (α)—the angle showing the dynamics of clot formation; MCF—a parameter reflecting the strength of the formed clot to resist the pin oscillation; and AUC—indicating the maximum clot formation. Coagulation was triggered by the addition of calcium chloride (12 mM final concentration) into samples and the analyses were performed at least 35 min, to the moment when all measured parameters were collected.

### 5.8. Aggregation in Whole Blood In Vitro

Collagen-stimulated platelet aggregation in whole blood was measured by the impedance method in a Chronolog aggregometer (Chrono-log Corporation, Havertown, PA, USA) according to the method previously described by Cardinal and Flower [41] with further modification of Kalaska et al. [42].

The following concentrations of IS were examined: 0.1 mM, 0.5 mM, and 1 mM. Stimulation of aggregation was performed using collagen in final concentration of 5 μg/mL. The results were expressed by four parameters: amplitude, slope, lag time of aggregation, and AUC. 

### 5.9. Aggregation in PRP In Vitro

PRP aggregation was assessed by the optical method in a Chronolog aggregometer (Chrono-log Corporation, Havertown, PA, USA) according to the method described by Dhurat et al. [43]. Stimulation of aggregation was performed by ADP (5 μM) or ADP (5 μM) with 5-HT (2.5 μM). The following concentrations of IS were examined: 0.1 mM, 0.5 mM, and 1 mM. The results were expressed by four parameters: % of aggregation, slope, lag time of aggregation, and AUC. 

### 5.10. Erythrocyte Osmotic Resistance Assay

For evaluation of the osmotic resistance of erythrocytes, blood samples were collected into EDTA-K_2_ test tubes from the rats left ventricle and were tested according to Hunter [44] in our modification [45]. The osmotic resistance test was performed directly after blood withdrawn. The RBCs were incubated for 30 min with different concentrations of IS (0.1 mM, 0.5 mM, and 1 mM). In the control group the RBCs were incubated with PBS. Next, 10 μL of blood was added to 2 mL saline solution in range of concentration from 10 to 150 mM giving final osmolarity from 20 to 300 mOsm. The suspension was allowed to stand at room temperature for 30 min and was centrifuged at 1000 *g* for 5 min at 4 °C. The absorbance of received supernatants was determined in microplate reader (Dynex Tech., Chantilly, VA, USA) at 540 nm. Obtained values are presented as % of hemolysis.

### 5.11. Evaluation of Hematological Parameters

Evaluation of hematological parameters: WBC, RBCs, HGB, HCT, MCV, MCH, MCHC, PLT in blood samples taken from the rats left ventricle after iv administration of different IS doses (3, 10, 30 or 100 mg/kg b.w.) or 0.9% natrium chloride (control group) and collected into EDTA-K_2_ test tubes was assessed with an Animal Blood Counter (ABC Vet, Horiba, Viernheim, Germany) according to the manufacturer directions.

### 5.12. Statistical Analysis

The normally distributed data were presented as mean ± SD, while the non-Gaussian data as median (full-range). Normality of distribution was tested using Shapiro-Wilk *W* test. The Student *t* test or nonparametric Mann-Whitney test were used to compare differences between experimental groups and control group. A two-tailed *p* < 0.05 was considered to indicate significance. Computations were performed using GraphPad 6 Prism (GraphPad Software, La Jolla, CA, USA).

## Figures and Tables

**Table 1:** Impact of IS on coagulation parameters.

Parameter	VEH	10 (mg/kg b.w.)	30 (mg/kg b.w.)	100 (mg/kg b.w.)
**APTT (s)**	24.30 ± 1.70	20.4 ± 1.50 ***	19.1 ± 1.2 ***	19.7 ± 1.3 ***
**Fibrinogen (g/L)**	2.27 ± 0.06	2.18 ± 0.07 *	2.15 ± 0.04 **	2.19 ± 0.08 *
**PT (s)**	10.50 ± 0.60	11.40 ± 0.40 *	10.90 ± 0.90 NS	10.5 ± 0.90 NS
**TT (s)**	34.40 ± 2.10	36.00 ± 4.50 NS	37.40 ± 3.50 NS	33.3 ± 1.40 NS

APTT—activated partial thromboplastin time; IS—indoxyl sulfate; PT—prothrombin time; NS—non-significant; TT—thrombin time; VEH—control group; * *p* < 0.05; ** *p* < 0.01; *** *p* < 0.001.

**Table 2:** Parameters of dynamic of clot formation in whole blood after exposure to IS.

Parameter	VEH	10 (mg/kg b.w.)	30 (mg/kg b.w.)	100 (mg/kg b.w.)
**Alpha (angle)**	70 ± 3.6	67.7 ± 2.8 NS	68.4 ± 2.6 NS	71 ± 6.3 NS
**AUC (a.u.)**	6661 ± 337	6761 ± 202 NS	6764 ± 220 NS	7231 ± 642 *
**CT (s)**	382 (333–477)	391 (306–479) NS	392 (234–427) NS	323 (220–332) **^,##,^^
**MCF (mm)**	66.1 ± 2.8	67.5 ± 3.3 NS	67.2 ± 2.2 NS	73.3 ± 6.3 *^,#,^^
**CFT (s)**	129 ± 20.6	127 ± 20.5 NS	113 ± 12.7 NS	88.6 ± 30.6 *

IS—indoxyl sulfate; VEH—control group; AUC—area under the curve; CT—clotting time; MCF—maximal clot firmness; CFT—clot formation time; NS—non-significant; * *p* < 0.05 compared to control; ** *p* < 0.01 compared to control; ^#^ *p* < 0.05 compared to 10 mg. b.w. of IS; ^##^ *p* < 0.01 compared to 10 mg/kg b.w. of IS; ^^^ *p* < 0.05 compared to 30 mg/kg b.w. of IS.

**Table 3:** Impact of IS on ADP- and ADP+ 5-HT-induced platelet aggregation in platelet-rich plasma.

Parameter	ADP				ADP+ 5-HT			
	VEH	0.1 (mmol)	0.5 (mmol)	1.0 (mmol)	VEH	0.1 (mmol)	0.5 (mmol)	1.0 (mmol)
**AUC (a.u.)**	75.7 ± 34.1	73.2 ± 19.8 NS	85.2 ± 34.8 NS	89 ± 29.8 NS	131 ± 13.1	130 ± 17.0 NS	133 ± 11.6 NS	122 ± 17.7 NS
**Lag Time (s)**	36.9 ± 18.8	43.3 ± 7.9 NS	39.4 ± 19.2 NS	40.8 ± 16.8 NS	21.3 ± 5.85	18.6 ± 4.21 NS	19.4 ± 2.13 NS	20.9 ± 6.24 NS
**Percent (%)**	49.3 ± 10.4	47.2 ± 10.4 NS	49.3 ± 14.9 NS	53.1 ± 8.1 NS	64.4 ± 3.20	62.0 ± 6.23 NS	65.4 ± 5.37 NS	61.9 ± 6.31 NS
**Slope (a.u.)**	31 ± 6.1	30.8 ± 4.9 NS	32.7 ± 7.1 NS	35 ± 7.1 NS	38.5 ± 1.77	38.9 ± 1.73 NS	39.1 ± 2.53 NS	36.6 ± 2.26 NS

IS—indoxyl sulfate; VEH—control group; 5-HT—serotonin; ADP—adenosine diphosphate; a.u.—arbitral units; NS—non-significant; s—seconds.

**Table 4:** Impact of IS on hematological parameters.

Parameter	VEH	3 (mg/kg b.w.)	10 (mg/kg b.w.)	30 (mg/kg b.w.)	100 (mg/kg b.w.)
HCT (%)	41.7 ± 2.9	41 ± 3.1 NS	41 ± 2.8 NS	40.1 ± 2.1 NS	40.4 ± 2.3 NS
HGB (g/dL)	14.2 ± 0.7	13.8 ± 1.1 NS	13.7 ± 0.9 NS	13.5 ± 0.7 NS	13.4 ± 0.9 NS
MCH (pg)	19.5 ± 0.9	18.9 ± 0.7 NS	19.1 ± 0.7 NS	19.4 ± 0.8 NS	19 ± 1.1 NS
MCHC (g/dL)	33.8 ± 0.9	33.3 ± 0.3 NS	33 ± 0.8 NS	33.5 ± 0.9 NS	33.2 ± 0.6 NS
MCV (μm^3^)	56.6 ± 2.1	56.6 ± 1.3 NS	57.4 ± 1.1 NS	57.6 ± 1.8 NS	57.4 ± 1.9 NS
PLT (10^3^/mm^3^)	482 ± 48.3	498 ± 36 NS	526 ± 37.4 NS	506 ± 42.8 NS	519 ± 43.5 NS
RBC (10^6^/mm^3^)	7.3 ± 0.7	7.2 ± 0.6 NS	7.1 ± 0.5 NS	7 ± 0.4 NS	7.1 ± 0.4 NS
WBC (10^3^/mm^3^)	2.4 (1.2–4.2)	1.8 (1.5–3.1) NS	1.9 (1.3–3) NS	2.1 (1.1–2.7) NS	2.3 (1.9–2.9) NS

HCT—hematocrit; HGB—hemoglobin; IS—indoxyl sulfate; MCH—mean corpuscular hemoglobin; MCHC—mean corpuscular hemoglobin concentration; MCV—mean corpuscular volume; NS—non-significant; PLT—platelet; RBC—red blood cells; WBC—white blood cells; VEH—control group.
